# The comparative anti-oxidant and anti-inflammatory efficacy of postbiotics and probiotics through Nrf-2 and NF-kB pathways in DSS-induced colitis model

**DOI:** 10.1038/s41598-024-62441-0

**Published:** 2024-05-21

**Authors:** Niloofar Rezaie, Shadi Aghamohammad, Elham Haj Agha Gholizadeh Khiavi, Shohreh Khatami, Aria Sohrabi, Mahdi Rohani

**Affiliations:** 1 Department of Bacteriology, Pasteur Institute of Iran, Tehran, Iran; 2 Department of Biochemistry, Pasteur Institute of Iran, Tehran, Iran; 3 Department of Epidemiology and Biostatistics, Research Centre for Emerging and Re-Emerging Infectious Diseases, Pasteur Institute of Iran, Tehran, Iran

**Keywords:** Probiotic, Postbiotic, Oxidative stress, Inflammation, Microbiology, Diseases

## Abstract

IBD is a disorder which could be caused by oxidative stress. This investigation aims to determine if probiotics and postbiotics can control oxidative stress and inflammation and compare the effectiveness of these two probiotic and postbiotic mixtures of substances. 88 strains of *Lactobacillus* and *Bifidobacterium* were tested for antioxidant activity. Male wild-type C57BL/6 mice were divided into four experimental groups, namely high fat diet (HFD) + PBS, HFD + DSS, HFD + DSS + 10^9^ cfu/ml of probiotics, and HFD + DSS + 10^9^ cfu/ml of postbiotics. The phenotypical indices and pathological scores were assessed. The expression of genes related to NF-kB and Nrf2 signaling pathways and enzymes associated with oxidant/anti-oxidant activities, and proinflammatory/inflammatory cytokines were assessed. In contrast to the groups exposed to DSS, mice treated with probiotics mixture and postbiotics mixture alongside DSS displayed alleviation of DSS-induced adverse effects on phenotypical characteristics, as well as molecular indices such as the Nrf2 and NF-kB related genes, with a greater emphasis on the postbiotics component. In accordance with the findings of the present investigation, it can be inferred that even in using a high-fat dietary regimen as an inducer of oxidative stress, the emergence of inflammation can be effectively addressed through the utilization of probiotics and, more specifically, postbiotics.

## Introduction

The concept of oxidative stress, which refers to an imbalance between the generation of reactive oxygen species (free radicals) and the body's antioxidant defenses, is examined in the context of its potential contribution to the development of tissue damage in various abnormal condition, including Inflammatory Bowel Disease (IBD). Although IBD encompasses various etiological factors, it is noteworthy that prolonged and uncontrolled oxidative stress can also contribute to the onset and development of IBD^1^. Oxidative stress could initiate IBD via various mechanisms. On one hand, the presence of increased oxidative stress in conjunction with the initiation of reactive aldehydes derived from lipid peroxidation (LDRAs) exhibits a strong correlation with the magnitude of autoimmune illness^2^. On the other hand, oxidative stress precipitates the impairment of the mucosal layer within the gastrointestinal tract and facilitates bacterial infiltration, thus triggering the immune system's defensive reaction and inciting the onset of IBD. Under normal physiological circumstances, cells have the capability to endure a specific amount of reactive oxygen species (ROS) due to their antioxidant capacity. This ability is of vital importance for the preservation of intestinal homeostasis. Nevertheless, an excessive load of oxidants, resulting from increased ROS production or reduced reduction reactions, can significantly enhance the permeability of cellular membranes. This increase in permeability is one of the primary factors contributing to the onset of inflammation^3^.

Concerning the role of gut microbiota on cell and intestinal permeability^4^, it could be said that gut microbiota could play important role in controlling oxidative stress and inflammation. The disruption of gut microbiota leads to an intricate interplay involving the mechanisms of inflammation, modulation of host defense, heightened oxidative stress, and changes in the metabolism derived from bacteria^5^. Since the appearance and progression of IBD are accompanied by enduring oxidative stress and inflammatory reactions provoked by an imbalanced gut microbiota^6^, the utilization of any substance capable of influencing dysbiosis and concurrently possessing antioxidant properties can be regarded as a crucial element in managing IBD.

Probiotics have been characterized by the Food and Agriculture Organization (FAO) and the World Health Organization (WHO) of the United Nations as "live microorganisms that, when administered in sufficient quantities, bestow a health advantage upon the host^7^. These microorganisms have the capacity to regulate the makeup of the gastrointestinal microbiota, enhance the structural soundness of tissues through their effect on tight junctions, and potentially mitigate inflammation^8^. In addition to these effects, probiotics have also been shown to possess antioxidant properties. These properties involve the removal of reactive oxygen species, the binding of metal ions to prevent the Fenton reaction, which is defined as the oxidation of organic contaminants by hydrogen peroxide and Fe (II), and the control of host antioxidant enzymes^9^. Henceforth, it can be postulated that the utilization of probiotics may serve as a prospective approach in regulating the detrimental consequences that manifest in IBD. Postbiotics, in addition to probiotics, have also been demonstrated to possess advantageous impacts on an individual's well-being. According to The International Scientific Association of Probiotics and Prebiotics (ISAPP), postbiotics are defined as inanimate microorganisms and/or their components that provide a health benefit to the host^10^. However, there are additional interpretations of postbiotics. In a different context, postbiotics are derived from fermentations primarily carried out by lactic acid bacteria and yeast. Postbiotics include substances such as short-chain fatty acids, bacteriocins, and organic acids. A centrifuge is utilized in one of the processes for producing postbiotics, separating the biomass from the cell-free supernatant^11^. It is of utmost importance to emphasize that the utilization of postbiotic agents possesses the potential to effectively mitigate safety concerns among individuals afflicted with augmented intestinal permeability and compromised immune systems^12^.

Since IBD is among the ailments that have the potential to impact the quality of life of individuals, and even intensify in individuals adhering to an improper dietary regimen, such as high fat diet (HFD) through increasing oxidative stress^13^, it becomes imperative to incorporate beneficial compounds such as probiotics and postbiotics. The objective of this investigation is to address the query of whether probiotics and postbiotics can possess distinct anti-oxidative properties in the presence of a high-fat diet and mitigate inflammation. Additionally, it aims to determine the superior efficacy between these two substances.

## Results

### Results of in-vitro analysis

#### The antioxidant capacities and ability of scavenge free radicals among our native probiotic strains and native postbiotics

As said above six strains, including *Lactobacillus* reuteri RP100, *Lactobacillus plantarum* RP42, *Lactobacillus plantarum* RP119, *Lactobacillus plantarum* RP155, *Bifidobacterium bifidum* RP1001, and *Bifidobacterium longum* RP1044 with the highest antioxidant activity were chosen. The capabilities of the strains for DPPH varied from 52.33% (*B. longum* RP1044) to 72.67% (*L. plantarum* RP42) and for ABTS 50.33% (*B. bifidum* RP1001) to 66.67% (*L. reuterri* RP100). Hydroxyl Radical Scavenging (HRS) test in this specific subset showcased a range of activity, ranging from 50% for *B. bifidum* RP1001 to 70% for *L. reuteri* RP100. *L. reuteri* RP100 exhibited a level of activity amounting to 68%, whereas *B. longum* 1044 displayed an activity level of 50.2%, concerning to the Superoxide Anion Assays. *L. plantarum* RP155 with values of 3.46 and *L. plantarum* RP42 with value of 2.95 showcased the highest and lowest levels of activity in the RP test. In the Lipid Peroxidation Inhibition test, *B. longum* RP1044 and *L. reuteri* RP100 demonstrated activity levels of 53% and 65%, respectively. Additionally, it is worth mentioning that the antioxidant capacity of our probiotic cocktail was significantly superior to that of each individual probiotic strain (see Supplementary file 1).

The levels of antioxidant activity observed in our native postbiotics were remarkably high. The strains exhibited varying abilities in terms of DPPH, ranging from 77% (*L. plantarum* RP155) to 81% (*L. plantarum* RP119), and for ABTS, from 78.6% (*B. longum* RP1044) to 80% (*L. reuterri* RP100). Within this specific subset, the Hydroxyl Radical Scavenging (HRS) test demonstrated a range of activity, spanning from 71.3% for *L. plantarum* RP119 to 77.3% for *L. reuteri* RP100. *B. bifidum* RP1001 exhibited a level of activity reaching 79%, while *B. longum* RP1044 displayed an activity level of 72% in relation to the Superoxide Anion Assays. Notably, the RP test revealed that *B. longum* RP1044 and *L. plantarum* RP42 exhibited the highest and lowest levels of activity, with values of 3.55 and 3.37, respectively. In the Lipid Peroxidation Inhibition test, *B. longum* RP1044 and B. bifidum RP1001 demonstrated activity levels of 77.3% and 72.6%, respectively. Furthermore, it is essential to highlight that the antioxidant capacity of our postbiotic mixture was significantly superior to that of each individual probiotic strain (see Supplementary file 1).

#### Identification of volatile compounds in postbiotics using gas chromatography–mass spectrometry

The GC–MS analysis revealed Acetic acid, hexanol, and Pyrogallol as antioxidants present in the postbiotic cocktail. Particularly, acetic acid, known as a short-chain fatty acid, displayed significant promise as an antioxidant agent.

### The results of in-vivo experiments

#### The results of weight gain, DAI, intestinal length and histopathological scores

**Figure 1:**
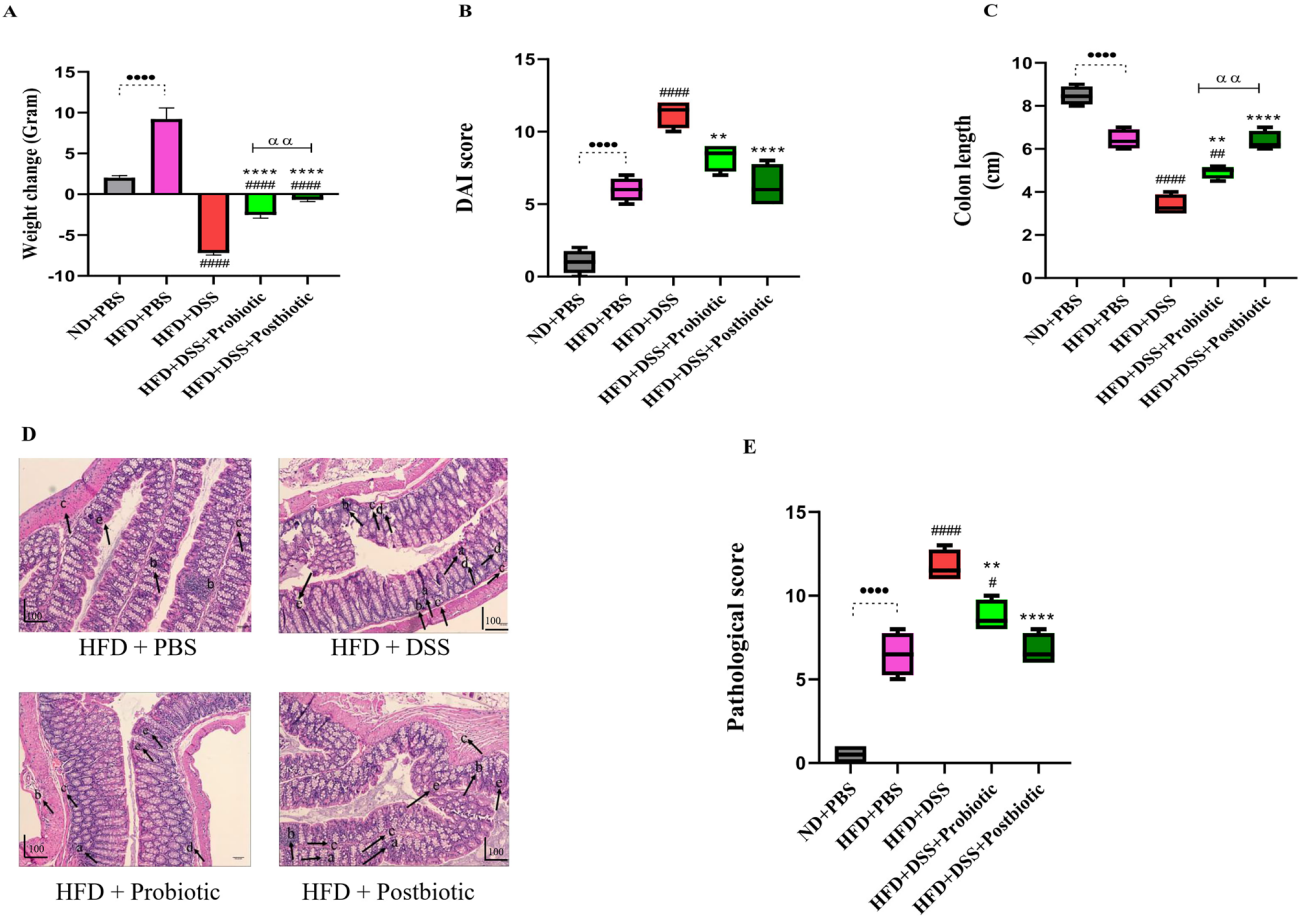
Effects of probiotics and postbiotics mixture on disease severity in DSS-induced colitis mice. (**A**) Body weight changes, (**B**) DAI score, (**C**) Colon length, (**D**) H&E staining of colon section of mice (a: crypts architecture, b: inflammation, c: muscle thickness, d: goblet cells depletion, and e: crypts abscesses, The scale bar is 100 pixels. (**E**) histopathological score. Data are presented as the mean ± SD, N = 5 per group. Statistical significance was determined using the following symbols: ^●^, p < 0.05; ^●●^, p < 0.01; ^●●●^, p < 0.001; ^●●●●^, p < 0.0001 (ND + PBS and HFD + PBS), ^#^, p < 0.05; ^##^, p < 0.01; ^###^, p < 0.001; ^####^, p < 0.0001 (HFD + PBS vs. Other groups), *, p < 0.05; **, p < 0.01; ***, p < 0.001; ****, p < 0.0001 (HFD + DSS vs. Other groups), ^α^, p < 0.05; ^αα^, p < 0.01; ^ααα^, p < 0.001; ^αααα^, p < 0.0001, The relatedness between HFD + DSS + probiotic and HFD + DSS + postbiotic groups. HFD: 60%Kcal, Fat 35%, Protein 24%, Carbohydrate 26%, Calories 52kcal/gr + PBS, DSS: DSS (2%) + PBS, HFD + Probiotic: HDF + probiotic cocktail (10^9^ CFU), HFD + postbiotics (HDF + postbiotic cocktail (10^9^ CFU).

The findings pertaining to alterations in weight and intestinal length, as well as the corresponding criteria with regards to DAI and histopathological scale, are shown in Fig. 1. Based on our findings, the utilization of a High-Fat Diet (HFD) in comparison to the negative control group (ND + PBS) resulted in significant adverse effects on weight gain and colitis indices (p < 0.0001). According to our research results, the utilization of a HFD resulted in a significant increase in weight gain of nearly 10g over a period of 28 days. Remarkably, when mice underwent inflammation, induced by the administration of DSS, there was a substantial loss in weight of approximately 10 g (p < 0.0001). Interestingly, the implementation of our native probiotic strains and postbiotics demonstrated a significant impact on weight loss as compared to the DSS group (p < 0.0001). Furthermore, it is noteworthy that the utilization of our postbiotic cocktail led to a comparatively lesser degree of weight reduction (p < 0.05). The findings of our study revealed the favorable impacts of our native probiotic strains and postbiotics as compared to the DSS group. The administration of DSS resulted in a notable increase in the DAI score (p < 0.0001), whereas both the probiotic and postbiotic treatments (with no significant difference) were able to significantly reduce the aforementioned score (p < 0.01). Regarding the findings pertaining to colon length, our native probiotic strains and postbiotic agents demonstrated a notable positive impact, as they were able to enhance the length of the colon (p < 0.01). Furthermore, compared to probiotic strains, the effects of the postbiotic agents (p < 0.05) were particularly noteworthy compared to the group treated with DSS, which significantly contributed to a reduction in colon length (p < 0.0001). When considering the pathological score, the utilization of DSS demonstrates a substantial enhancement in the score (p < 0.0001). Conversely, our native probiotic strains and postbiotics exhibit a significant reduction in the score (p < 0.01), with no discernible disparity between the effects of probiotic and postbiotic interventions.

#### The results of antioxidant and oxidant enzymes in the serum and gut

**Figure 2:**
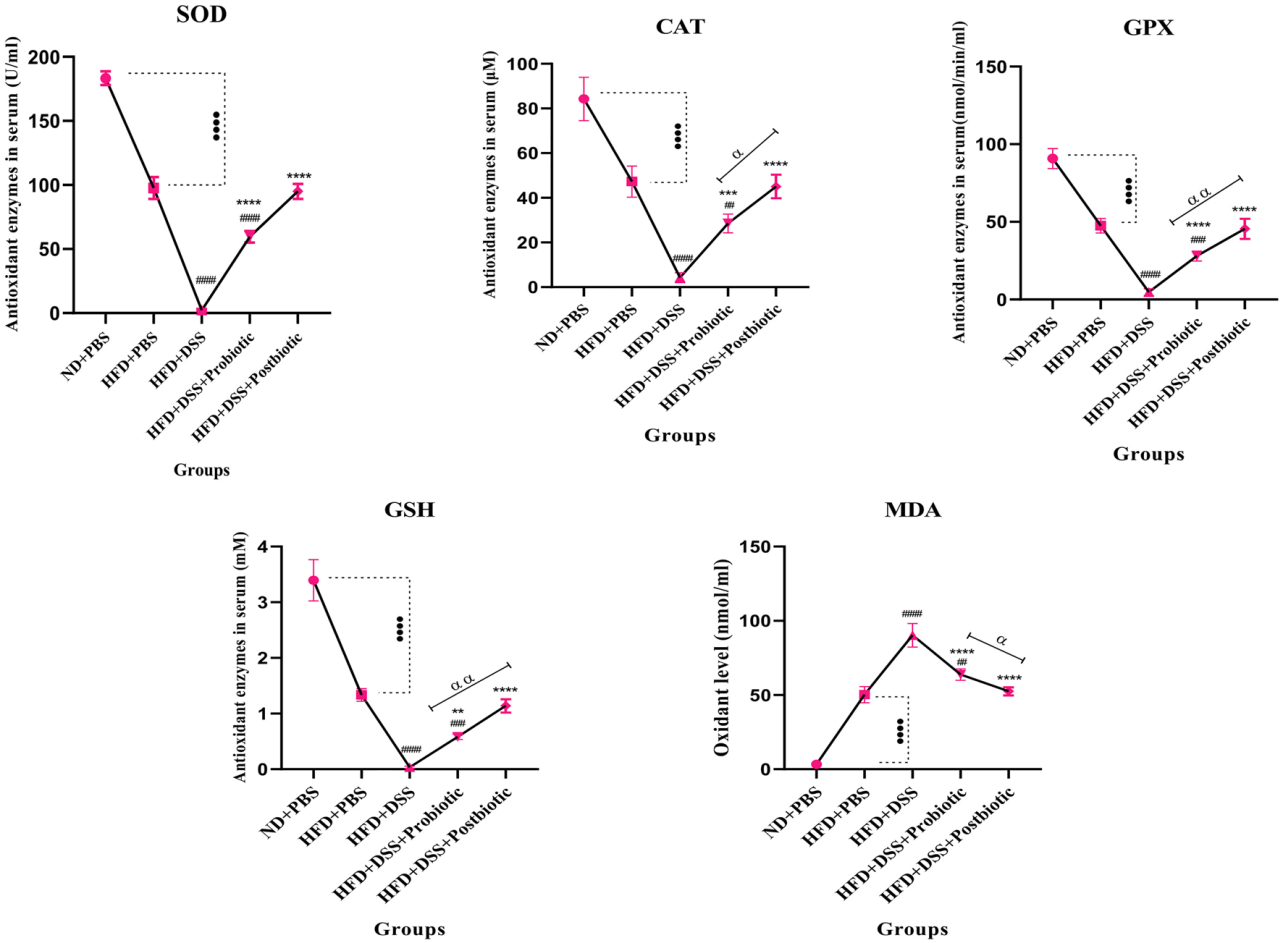
The levels of SOD, CAT, GSH, GPX (antioxidant enzymes), and MDA oxidant enzyme in serum. Data are presented as the mean ± SD, N = 5 per group. Statistical significance was determined using the following symbols: ^●^, p < 0.05; ^●●^, p < 0.01; ^●●●^, p < 0.001; ^●●●●^, p < 0.0001 (ND + PBS and HFD + PBS), ^#^, p < 0.05; ^##^, p < 0.01; ^###^, p < 0.001; ^####^, p < 0.0001 (HFD + PBS vs. Other groups), *, p < 0.05; **, p < 0.01; ***, p < 0.001; ****, p < 0.0001 (HFD + DSS vs. Other groups), ^α^, p < 0.05; ^αα^, p < 0.01; ^ααα^, p < 0.001; ^αααα^, p < 0.0001, The relatedness between HFD + DSS + probiotic and HFD + DSS + postbiotic groups. HFD: 60%Kcal, Fat 35%, Protein 24%, Carbohydrate 26%, Calories 52kcal/gr + PBS, DSS: DSS (2%) + PBS, HFD + Probiotic: HDF + probiotic cocktail (10^9^ CFU), HFD + postbiotics (HDF + postbiotic cocktail (10^9^ CFU).

**Figure 3:**
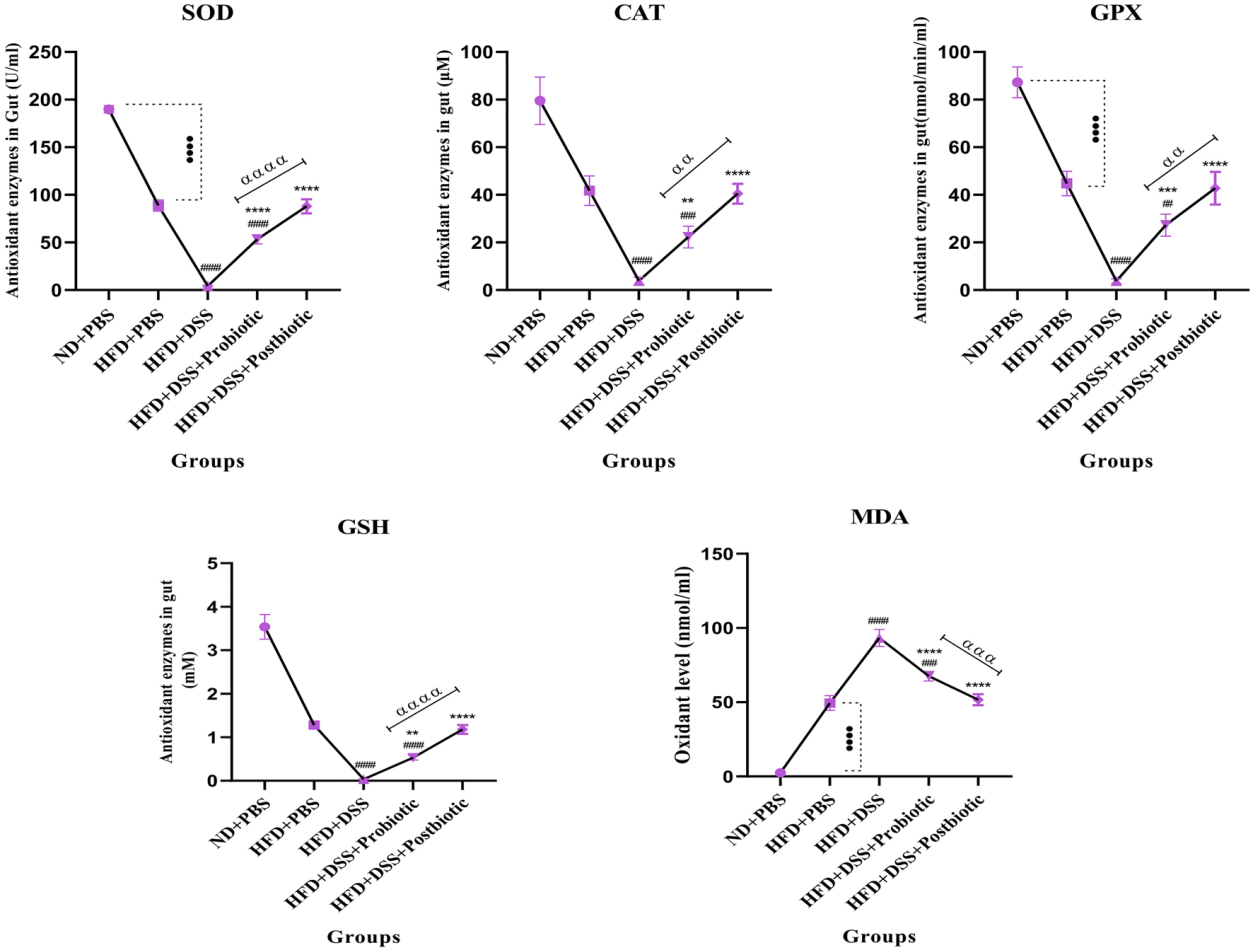
The levels of SOD, CAT, GSH, GPX (antioxidant enzymes), and MDA oxidant enzyme in gut. Data are presented as the mean ± SD, N = 5 per group. Statistical significance was determined using the following symbols: ^●^, p < 0.05; ^●●^, p < 0.01; ^●●●^, p < 0.001; ^●●●●^, p < 0.0001 (ND + PBS and HFD + PBS), ^#^, p < 0.05; ^##^, p < 0.01; ^###^, p < 0.001; ^####^, p < 0.0001 (HFD + PBS vs. Other groups), *, p < 0.05; **, p < 0.01; ***, p < 0.001; ****, p < 0.0001 (HFD + DSS vs. Other groups), ^α^, p < 0.05; ^αα^, p < 0.01; ^ααα^, p < 0.001; ^αααα^, p < 0.0001, The relatedness between HFD + DSS + probiotic and HFD + DSS + postbiotic groups. HFD: 60%Kcal, Fat 35%, Protein 24%, Carbohydrate 26%, Calories 52kcal/gr + PBS, DSS: DSS (2%) + PBS, HFD + Probiotic: HDF + probiotic cocktail (10^9^ CFU), HFD + postbiotics (HDF + postbiotic cocktail (10^9^ CFU).

The findings of the examination of antioxidant and oxidant enzymes in the bloodstream and gastrointestinal tract are visually represented in Figs. 2 and 3. Once more, our findings demonstrated the clear negative impact of HFD consumption on antioxidant indicators when compared to the negative control group (p < 0.0001). Based on our findings of serum, the use of DSS resulted in a reduction of the markers, such as SOD, CAT, GPX, and GSH, to nearly zero levels, while MDA levels increased following the utilization of DSS (p < 0.0001). Our native probiotics and postbiotic products were able to significantly enhance the levels of SOD, CAT, GPX, and GSH, while decreasing MDA levels (P < 0.0001). It is noteworthy that the effects of postbiotics on all markers, except for SOD, were more pronounced compared to our native probiotic strains (p < 0.05). The identical outcomes were replicated in relation to these markers within the gastrointestinal tract, albeit with the distinction that on this occasion, postbiotics exhibited greater efficacy than probiotics across all markers (p < 0.01).

#### The results of cytokines

**Figure 4:**
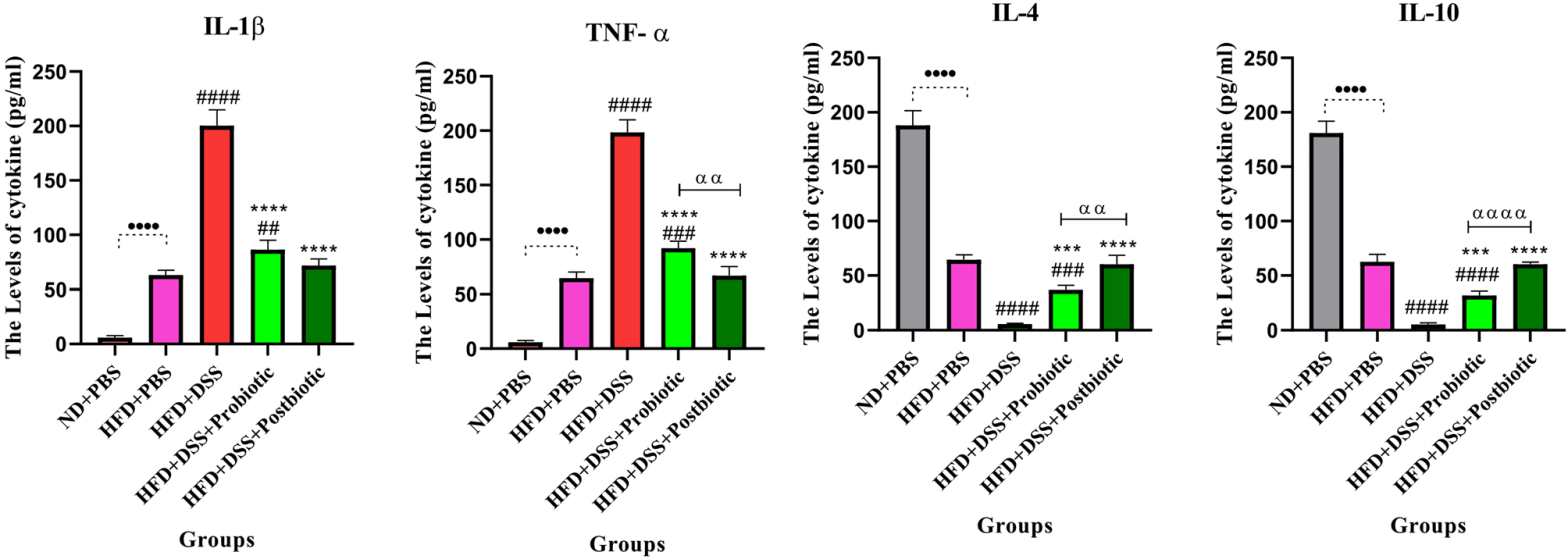
The Levels of IL-1β, TNF-α (Inflammatory cytokines) and, IL-4, IL-10 (anti-inflammatory cytokines), in serum. Data are presented as the mean ± SD, N = 5 per group. Statistical significance was determined using the following symbols: ^●^, p < 0.05; ^●●^, p < 0.01; ^●●●^, p < 0.001; ^●●●●^, p < 0.0001 (ND + PBS and HFD + PBS), ^#^, p < 0.05; ^##^, p < 0.01; ^###^, p < 0.001; ^####^, p < 0.0001 (HFD + PBS vs. Other groups), *, p < 0.05; **, p < 0.01; ***, p < 0.001; ****, p < 0.0001 (HFD + DSS vs. Other groups), ^α^, p < 0.05; ^αα^, p < 0.01; ^ααα^, p < 0.001; ^αααα^, p < 0.0001, The relatedness between HFD + DSS + probiotic and HFD + DSS + postbiotic groups. HFD: 60%Kcal, Fat 35%, Protein 24%, Carbohydrate 26%, Calories 52kcal/gr + PBS, DSS: DSS (2%) + PBS, HFD + Probiotic: HDF + probiotic cocktail (10^9^ CFU), HFD + postbiotic (HDF + postbiotic cocktail (10^9^ CFU).

Based on our research results, the utilization of a high-fat diet could have a notable impact on the levels of anti-inflammatory and pro-inflammatory cytokines when compared to the negative control (p < 0.0001). On the other hand, according to the findings of our study, the utilization of our native probiotic and postbiotic agents demonstrated a significant reduction in the levels of IL-1β and TNF-α, which are known to be pro-inflammatory cytokines (p < 0.0001). Conversely, the levels of IL-4 and IL-10, which are anti-inflammatory cytokines, were observed to increase significantly (p < 0.0001). These results were in contrast to the effects of DSS, which led to an increase in pro-inflammatory cytokines and a decrease in anti-inflammatory cytokines (p < 0.001). In general, the use of our postbiotic agents exhibited notable favorable effects, including a significant decrease in TNF-α levels (p < 0.01), as well as significant increases in IL-4 (p < 0.001) and IL-10 (p < 0.0001) levels compared to native probiotic strains (see Fig. 4).

#### The effects of our native probiotic strains on gene expression

**Figure 5:**
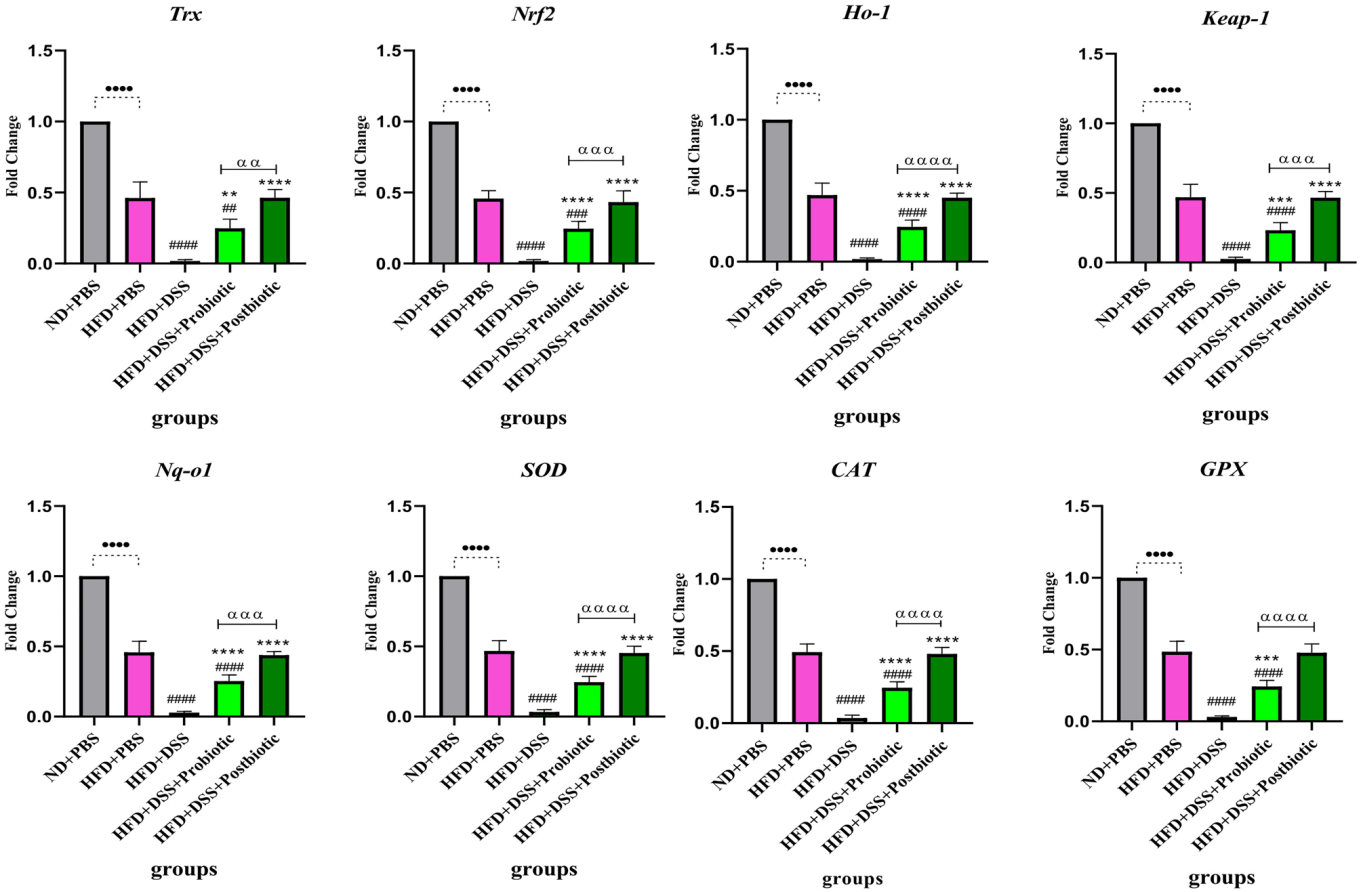
Relative gene expression [mean fold change] of antioxidants and Nrf2 related pathway genes expression in the different groups of treatments. Data were normalized with *gapdh*. Data are presented as the mean ± SD, N = 5 per group. Statistical significance was determined using the following symbols: ^●^, p < 0.05; ^●●^, p < 0.01; ^●●●^, p < 0.001; ^●●●●^, p < 0.0001 (ND + PBS and HFD + PBS), ^#^, p < 0.05; ^##^, p < 0.01; ^###^, p < 0.001; ^####^, p < 0.0001 (HFD + PBS vs. Other groups), *, p < 0.05; **, p < 0.01; ***, p < 0.001; ****, p < 0.0001 (HFD + DSS vs. Other groups), ^α^, p < 0.05; ^αα^, p < 0.01; ^ααα^, p < 0.001; ^αααα^, p < 0.0001, The relatedness between HFD + DSS + probiotic and HFD + DSS + postbiotic groups. HFD: 60%Kcal, Fat 35%, Protein 24%, Carbohydrate 26%, Calories 52kcal/gr + PBS, DSS: DSS (2%) + PBS, HFD + Probiotic: HDF + probiotic cocktail (10^9^ CFU), HFD + postbiotics (HDF + postbiotic cocktail (10^9^ CFU).

**Figure 6:**
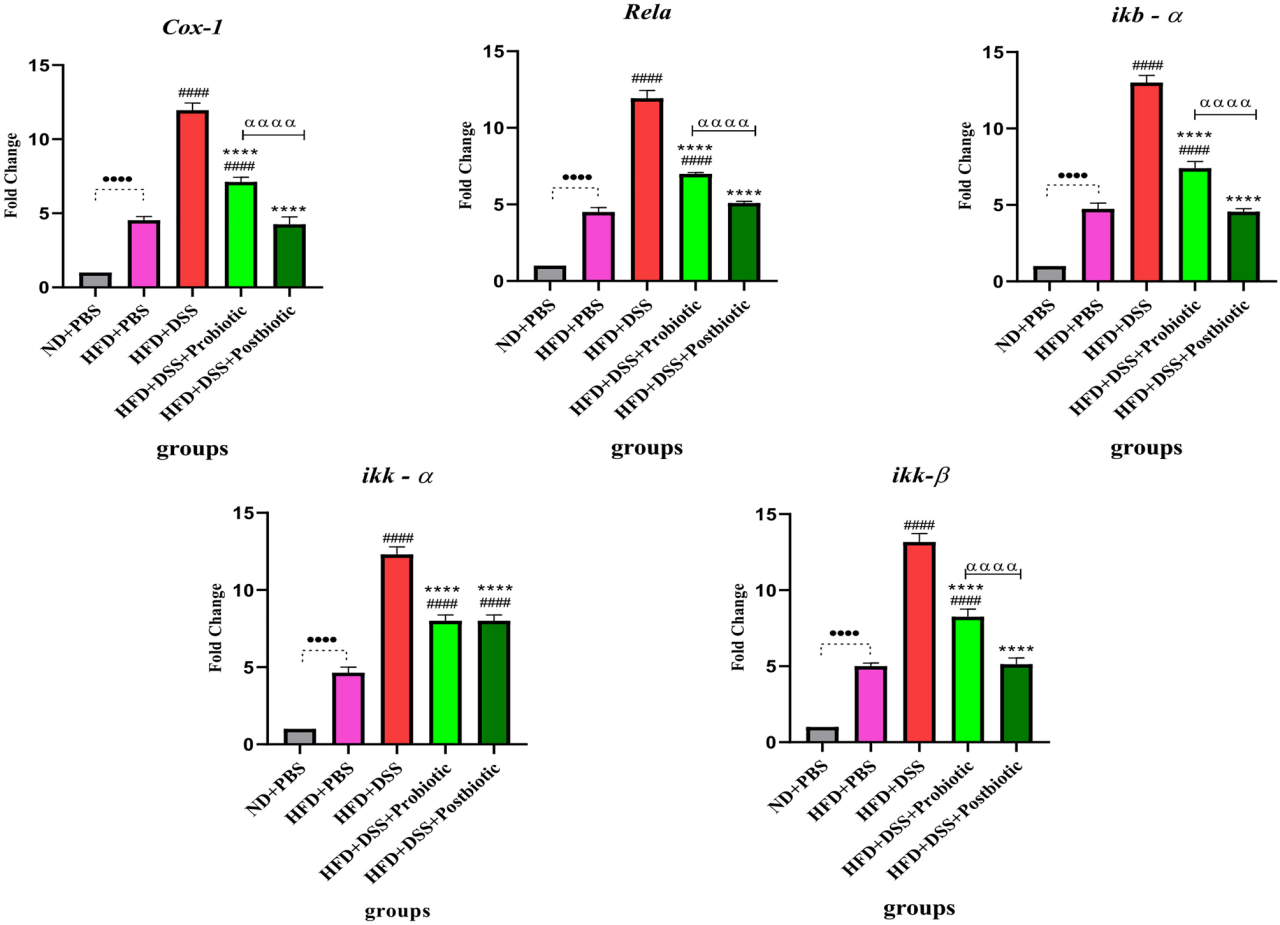
Relative gene expression [mean fold change] of NF-kB related pathway genes expression in the different groups of treatments. Data were normalized with *gapdh*. Data are presented as the mean ± SD, N = 5 per group. Statistical significance was determined using the following symbols: ^●^, p < 0.05; ^●●^, p < 0.01; ^●●●^, p < 0.001; ^●●●●^, p < 0.0001 (ND + PBS and HFD + PBS), ^#^, p < 0.05; ^##^, p < 0.01; ^###^, p < 0.001; ^####^, p < 0.0001 (HFD + PBS vs. Other groups), *, p < 0.05; **, p < 0.01; ***, p < 0.001; ****, p < 0.0001 (HFD + DSS vs. Other groups), ^α^, p < 0.05; ^αα^, p < 0.01; ^ααα^, p < 0.001; ^αααα^, p < 0.0001, The relatedness between HFD + DSS + probiotic and HFD + DSS + postbiotic groups. HFD: 60%Kcal, Fat 35%, Protein 24%, Carbohydrate 26%, Calories 52kcal/gr + PBS, DSS: DSS (2%) + PBS, HFD + Probiotic: HDF + probiotic cocktail (10^9^ CFU), HFD + postbiotics (HDF + postbiotic cocktail (10^9^ CFU).

The effects of our native probiotic strains and postbiotics on gene expression could be seen in Figs. 5 and 6. When comparing the negative and positive control groups, it could be seen that using HFD could significantly lead to alteration the expression level of genes enrolled in Nrf2 and NF-kB signaling pathways (p < 0.00010. On the other hand, the native probiotic strains, along with postbiotics, exhibited significant antioxidant and anti-inflammatory effects by affecting the Nrf2 and NF-kB signaling pathway. In terms of the Nrf2 signaling pathway, the use of DSS resulted in a decrease in the expression level of all genes (p < 0.0001). However, our native probiotic strains and postbiotics were able to significantly increase the expression level (p < 0.01) compared to the DSS group. Among all the genes studied in relation to the Nrf2 signaling pathway, our postbiotics had a significantly greater effect in increasing the expression level compared to our native probiotic strains (p < 0.01). Regarding the NF-kB genes, DSS caused a significant increase in the expression level of genes (p < 0.0001). However, our native probiotic strains and postbiotics were able to significantly decrease the expression level (p < 0.0001). Once again, the postbiotics had a more desirable effect (decrease in level) compared to the probiotic strains (p < 0.0001), except for *ikk—α*. It can be said that our postbiotics was almost able to restore the level of gene expression to that of the group without receiving DSS.

## Discussion

Engaging in an unhealthy routine such as consuming a diet high in fat may lead to detrimental consequences, encompassing obesity, hypertension, cardiovascular ailments, and even oxidative stress^14^. Oxidative stress per se might potentially exert a significant influence on the onset of diseases, such as cancer and diabetes. This can be attributed to the fact that reactive oxygen species (ROS) are typically generated as by-products of metabolic processes and should ideally be maintained at a relatively low concentration. However, when exposed to environmental stressors, the level of ROS increases, leading to the initiation of oxidative stress. This imbalance subsequently leads to detrimental consequences^15^. The combination of following a high-calorie diet that leads to obesity and adopting a sedentary lifestyle, which is common these days, has the potential to worsen this situation^16^. Additionally, the consumption of diets that are rich in fat is considered to be one of the primary stimuli for the development of inflammatory bowel disease. Specifically, the presence of saturated fat has been found to significantly contribute to the exacerbation of inflammation^17^, This is primarily due to the fact that high-fat diets induce oxidative stress within the protective lining of the colon, leading to an increase in permeability. Consequently, this compromised barrier function results in inflammation within the colon lining. Furthermore, it has been observed that high-fat diets have the ability to disrupt the normal functioning of the gut barrier by altering the composition of bile salts present in the lumen^13,18^. Taken collectively, each agent that exhibits anti-inflammatory and antioxidant properties may be crucial for utilization by patients with IBD, particularly those who experience an increase in body weight and adiposity. In recent years, there has been a growing focus on probiotics and the production of these bacteria, known as postbiotics. Consequently, the aim of the present investigation was to demonstrate the antioxidative and anti-inflammatory efficacy of our native probiotic strains and postbiotics in mice subjected to a high-fat diet, as well as to compare the effectiveness of these agents in order to determine which agent should be prioritized for use.

**Figure 7:**
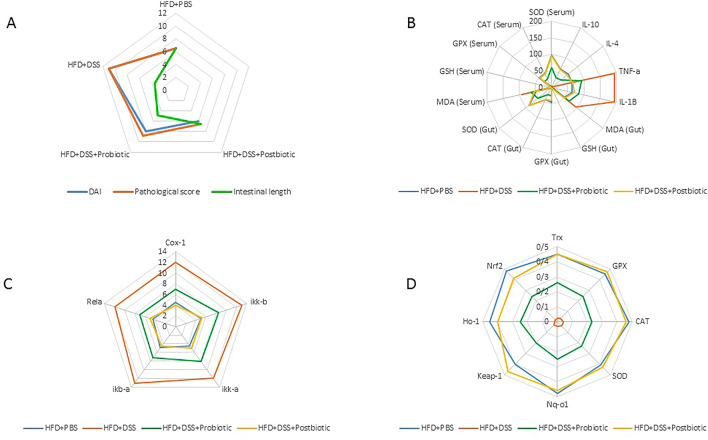
The general tendency of the present study is as follows: (**A**) the comprehensive outcomes of pathological assessment, (**B**) the comprehensive outcomes of phenotypic examinations, (**C**) the comprehensive outcomes of NF-kB manifestation, and (**D**) the comprehensive outcomes of Nrf2 manifestation.

The present study found that the use of a high-fat diet could worsen the situation by impacting both the molecular and phenotypical aspects of inflammation and oxidative stress. On the contrary both our native probiotic strains and postbiotics exhibited the potential for antioxidant activity through diverse assessments. The utilization of assays aimed at scavenging free radicals revealed that, regardless of the strain and type of test, even though both agents displayed antioxidant activity exceeding 50%, our postbiotics manifested a conspicuously greater level of activity. This level of activity was variable, varying from 50 to 72.6% in the case of our native probiotic strains, whereas our postbiotics exhibited activity within the range of 70.6% to 81%. The results of the in vivo experiments also confirmed the better effectiveness of our postbiotics. According to our results concerning the indices related to colitis our postbiotics could significantly had more anti-inflammatory effects compared with our native probiotic strains in body weight change and colon length. In other phenotypical assays, including the assessments of antioxidant markers in serum and gut and also cytokine measurement our native postbiotics could show completely more anti-inflammatory and anti-oxidant activities in compared to our native probiotic strains. When considering the findings at the molecular level, specifically the assessments of the Nrf2 and NF-kB pathways, it is apparent that our postbiotics exhibits a greater level of efficacy in comparison to our native probiotics. The findings of the current investigation are illustrated in Fig. 7. Broadly speaking, the present outcomes indicate that postbiotics exerted a comprehensive impact, encompassing both molecular and phenotypic dimensions. One explanation for the remarkable antioxidant effects of our native postbiotic may be attributed to the fact that our findings indicate acetic acid as the predominant component of the postbiotic. This compound is among the essential fatty acids capable of demonstrating significant antimicrobial and antioxidant properties^19^ This effect was substantial enough to restore the experimental conditions to the positive control state (HFD + PBS) and effectively suppress the deleterious consequences of DSS. In other words, when considering the collective evidence, it can be argued that while our native probiotic strains may yield advantageous outcomes, the incorporation of our postbiotics in conjunction with DSS has the potential to entirely counteract the detrimental impact of DSS, thereby resulting in a similar physiological state to that of the high-fat diet-fed group.

These data align with the findings of other studies. Kang et al., demonstrated that utilizing 17 distinct heat-killed isolates as postbiotics could exhibit both anti-inflammatory and anti-oxidant properties by conducting DPPH and ABTS assays. This can be attributed to the synthesis of short-chain fatty acids such as acetic, propionic, and butyric acid^20^. According to the findings of Li et al., the utilization of probiotic strains, such as *Lactobacillus delbrueckii*, *Lactobacillus fermentum*, and *Bacillus coagulans* TL3, resulted in the activation of the Nrf2/Ho-1 pathway. Consequently, this activation facilitated the confrontation of oxidative stress and the inhibition of ulcerative colitis^9^. Gao et al. demonstrate that the utilization of the probiotic *Saccharomyces boulardii* may potentially exhibit antioxidant efficacy by augmenting the levels of antioxidant enzymes (superoxide dismutase, catalase, and heme oxygenase 1) and glutathione, while concurrently reducing the accumulation of malondialdehyde^21^. Moreover, in accordance with the evidence provided by Liu et al., short chain fatty acids, which serve as the primary constituents of postbiotics, have the capacity to regulate the equilibrium between Th17 and Treg cells in order to alleviate inflammation through the Nrf2/HO-1 pathway^22^.

## Conclusion

In the realm of academic exploration, there exists a dearth of comprehensive and extensive investigations pertaining to the potential antioxidant properties exhibited by probiotics, a group of microorganisms that have been extensively studied for their beneficial health effects, and more specifically, postbiotics, which are the bioactive compounds produced by probiotics. Therefore, the current study endeavors to address this knowledge gap by presenting a comprehensive analysis that encompasses both phenotypic and molecular aspects. The aim is to demonstrate that even in the context of a high-fat diet, when inflammation arises, probiotics and specifically postbiotics possess the capacity to combat inflammation through mechanisms that involve their inherent antioxidant properties. This finding is of utmost significance, particularly for individuals afflicted with inflammatory bowel disease, as their lifestyle choices in contemporary times may contribute to the development and progression of this condition.

## Materials and methods

### In vitro assessments

#### Bacterial culture and cocktails preparation

A total of 88 native probiotic strains, including the strains comprised *L. plantarum, L. reuteri, L. casei, L. rhamnosus, L. mucosae, L. fermentum, L. delbrueckii,* and *L. brevis,* along with *B. bifidum, B. longum,* and *B. infantis* were isolated from the breast milk and stool samples of healthy Iranian individuals^23,24^. The specified strains were cultivated in MRS broth (Quelab, Canada) containing 0.5% L-cysteine under anaerobic conditions at a temperature of 37 °C for a duration of 20 h. The bacterial suspension was meticulously prepared and subsequently centrifuged at a speed of 12,000 rpm for a duration of 5 min at a temperature of 4 °C. Subsequently, the growth reached an optical density of 600, at which point each pellet was subjected to two rounds of washing with PBS. Each bacterial suspension, with a concentration of 10^9^ bacterial CFU/mL, was then subjected to a temperature of 100 °C for a duration of 10 min. Following this, the supernatant was collected and subjected to filtration using a 0.22 μm syringe filter (Millex^®^GS, Millipore). Both the probiotic cocktail and the postbiotic cocktail were stored at a temperature of − 80 °C until they were utilized. It is important to emphasize that in the preparation of a postbiotic cocktail and probiotic cocktail, the inoculation method has been executed in accordance with the single strain preparation approach, whereby all chosen strains are introduced into a common media container with equal quantities of each strain.

#### Gas chromatography (GC–MS) of postbiotics

The method was conducted according to Kam et al. with slight modifications^25^. Analysis of organic acids was carried out using a gas chromatography-mass spectrometer from Leco Corp. located in St. Josef, MI, USA. A 30 m DB-FATWAX-UI chromatographic column from Agilent Technologies in Santa Clara, CA was used, with high-purity helium as the carrier gas. Each sample (postbiotic cocktail), measuring 1 μL, was injected in splitless mode at a temperature of 250 °C. The program started at 40 °C for 2 min, followed by an increase of 7 °C/min up to 165 °C, 25 °C/min up to 240 °C, and a 5-min maintenance period. Electron impact ionization at 1329 eV was applied, with the ion source temperature set at 250 °C. The mass range was from 40 to 300 m/z, using an extraction frequency of 50 kHz and an acquisition rate of 200 spectra/s.

#### The assessment of antioxidant activity of our native probiotic strains and postbiotics

To evaluate the antioxidant efficacy of our native probiotic strains, the bacterial stocks was cultured in MRS broth at a temperature of 37 °C for a duration of 18 h. Following this, we obtained live bacterial cell pellets by subjecting them to centrifugation (at a speed of 8000*g* for a period of 10 min) at a temperature of 4 °C. These pellets were then washed twice using PBS. The assessment of antioxidant activity involved a three-step screening process that employed biochemical assays. From a total of 88 strains were subjected to screening through DPPH and ABTS scavenging activity tests. Subsequently, the second phase involved examining 44 strains that exhibited the highest activity in the first stage, using Superoxide anion and Hydroxyl Radical Scavenging activity tests. In the third phase, 22 strains, which were selected based on the results of the second stage, underwent screening through Reducing Power and lipid peroxidation inhibition tests. Finally, six strains, including *Lactobacillus reuteri* RP100, *Lactobacillus plantarum* RP42, *Lactobacillus plantarum* RP119, *Lactobacillus plantarum* RP155, *Bifidobacterium bifidum* RP1001, and *Bifidobacterium longum* RP1044 with the highest antioxidant activity were chosen. The antioxidant activity of these six strains was assessed using the identical biochemical approach for postbiotics. It is important to mention that MRS broth was utilized as the Blank in all of the experiments.

### In vivo experiment

A group of twenty-four male C57BL/6 mice, ranging in age from 4 to 6 weeks and weighing 16 g, were obtained from the Pasteur Institute of Iran (Karaj). These mice were housed in polycarbonate cages (each cage accommodating four mice). They were kept in a controlled environment with a temperature of 22 °C and a humidity of 50%, following a 12-h light/dark cycle. The mice had access to sterilized water and food, and underwent a 2-week period of adjustment to a standard normal diet. After this period, the mice, now 8 weeks old, were subjected to an exclusive High-Fat Diet (HFD) regimen for a duration of 28 days. The HFD provided 60% of total calories, with 35% from fat, 24% from protein, and 26% from carbohydrates, resulting in a caloric density of 52 kcal/g. In the third week of the HFD regimen, after experiencing weight gain, specific subgroups within the HFD category received distinct oral gavage treatments on a daily basis for a consecutive two-week period. The treatments consisted of the following: (1) A HFD in combination with 200 µl of PBS (referred to as HFD + PBS), (2) A HFD in combination with 200 µl of 2% dextran sulfate sodium (DSS) (referred to as HFD + DSS), (3) HFD in combination with 200 µl of a mixture containing 2% DSS and 10^9^ cfu/ml of our native postbiotic cocktail, and (4) A HFD in combination with 200 µl of a mixture containing 2% DSS and 10^9^ cfu/ml of our native probiotic cocktail. It is important to highlight that in order to assess the adverse impacts of employing a high-fat diet on pathological and molecular parameters, the HFD + DSS group (referred to as the positive control for oxidative stress induction) was compared with the group of mice that were nourished with a standard diet and PBS (referred to as the negative control for oxidative stress induction).

#### Sample collection

All mice involved in this experiment were subjected to cervical dislocation in order to achieve euthanasia. Following this procedure, the colon of each mouse was removed and thoroughly cleansed with PBS. The length of the colon, which acts as an indirect measure of the severity of the inflammatory response, was evaluated. Subsequently, the colon was divided into three distinct segments. One of these segments was used for histopathological examination, while the remaining two segments of colon tissue samples were homogenized to extract RNA. Additionally, each mouse in all groups underwent an assessment of body weight, fecal consistency, and the occurrence of significant hemorrhaging on a daily basis. It is important to note that all evaluations were carried out in a blinded manner. All experimental procedures strictly adhered to the ethical standards articulated in the Helsinki Declaration and received approval from the Animal Experimentation Committee of the Pasteur Institute of Iran (IR.PII.REC1400.061) for the ethical care and utilization of laboratory mice.

#### The analysis of disease activity index (DAI)

The assessment of the Disease Activity Index (DAI) involves the examination of a combination of factors, including weight loss, stool consistency, and stool bleeding, as described by Kwon et al.^26^. The combination of these three parameters is then calculated to obtain an overall clinical score. The assessment was carried out in a manner that ensured the evaluators were uninformed of the specifics.

#### Histopathological analysis

The examination of colon tissues fixed with paraformaldehyde, embedded in paraffin, and divided into 4 µm thick sections was conducted as part of the histopathological analysis. The paraffin sections were then dewaxed and stained using hematoxylin and eosin. The histological score was determined based on the criteria outlined in a previous study^27^.

#### The assessment of oxidative stress and antioxidant markers in intestinal tissue and serum

The levels of Malondialdehyde (MDA), glutathione (GSH), catalase (CAT), superoxide dismutase (SOD), and glutathione peroxidase (GPX) in serum and portions of the distal intestinal tissue were evaluated in accordance with the instructions provided by the manufacturer (Navand Salamat, Iran).

#### Evaluation of pro-inflammatory and anti-inflammatory cytokines in serum

The levels of IL-1β and TNF-α as proinflammatory cytokines as well as IL-4 and IL-10 as anti-inflammatory cytokines were evaluated by the ELISA kit in accordance with the instructions stipulated by the manufacturer (Karmania Pars Gene, Iran). According to the kit information, the standards and samples were introduced into the ELISA vials and left to incubate for 1 h. Subsequently, the vials underwent three washes and conjugation, followed by the addition of the antibody. After another hour of incubation, they were washed again. Next, HRP-avidin conjugation was introduced, followed by a 30-min incubation and wash. The substrate was then added and allowed to incubate for 15 min. The reaction was halted with a stopping solution, and the optical density (OD) was recorded at 450 nm.

#### cDNA synthesis and real-time quantitative polymerase chain reaction (RT-qPCR)

The RNA extraction from the colon of mice was conducted using the RNeasy Mini Kit, manufactured by Favorgen Biotech Corp in Taiwan, in accordance with the protocol provided by the manufacturer. The amount of RNA that was extracted was assessed utilizing NanoDrop. The purity, determined by the ratios A260/A280 and A260/230, was used to evaluate the quality. For the reverse transcription of RNA into cDNA, the cDNA synthesis kit, manufactured by Yekta Tajhiz Azma Co in Iran, was employed. The quantitative PCR (qPCR) was carried out using the ABI Prism 7900HT instrument with the utilization of 2× SYBR-Green, specifically the RealQ Plus Master Mix Green manufactured by Amplicon A/S in Denmark. All primer sequences can be found in Table 1.

**Table 1:** The primers used in the current study.

Genes	Primer sequence (5ʹ > 3ʹ)	Product size (bp)
*Nrf2MF*	TAGATGACCATGAGTCGCTTGC	153
*Nrf2MR*	GCCAAACTTGCTCCATGTCC	
*Keap1 MF*	TCGAAGGCATCCACCCTAAG	135
*Keap1MR*	CTCGAACCACGCTGTCAATCT	
*NQO1MF*	AGGATGGGAGGTACTCGAATC	127
*NQO1MR*	TGCTAGAGATGACTCGGAAGG	
*HO-1MF*	GGTGATGGCTTCCTTGTACC	155
*HO-1MR*	AGTGAGGCCCATACCAGAAG	
*Trx-1MF*	CTTTTGCCCGTCTCTCAATCA	181
*Trx-1MR*	AGGGTATTTCACACTTAGGTCCT	
*SOD2MF*	CAGACCTGCCTTACGACTATGG	113
*SOD2MR*	CTCGGTGGCGTTGAGATTGTT	
*CATMF*	GGAGGCGGGAACCCAATAG	102
*CATMR*	GTGTGCCATCTCGTCAGTGAA	
*Gpx1MF*	CCACCGTGTATGCCTTCTCC	105
*Gpx1MR*	AGAGAGACGCGACATTCTCAAT	
*COX-2(PTGS2) MF*	TGCACTATGGTTACAAAAGCTGG	271
*COX-2(PTGS2) MR*	TCAGGAAGCTCCTTATTTCCCTT	
*NF-kBp65(Rela) MF*	TGACCCCTGTCCTCTCACATCCG	94
*NF-kBp65(Rela) MR*	CAGCTCCCAGAGTTCCGGTT	
*NF-KBIA(IkBa)MF*	TGAAGGACGAGGAGTACGAGC	127
*NF-KBIA(IkBa)MR*	TGCAGGAACGAGTCTCCGT	
*Ikka (Chuk)MF*	GAGAGCGATGGTGCCATGAA	136
*Ikka (Chuk)MR*	CCAGAACAGTACTCCATTGCCAGA	
*Ikkb (IKBKB)MF*	AAGTACACCGTGACCGTTGAC	91
*Ikkb (IKBKB)MR*	GCTGCCAGTTAGGGAGGAA	
*GAPDHMF*	TGGCCTTCCGTGTTCCTAC	178
*GAPDHMR*	GAGTTGCTGTTGAAGTCGCA	

### Statistical analysis

The housekeeping gene *gapdh* was utilized for normalization purposes. The relative quantification of targeted gene expression was determined using the 2^−ΔΔCt^ method. The statistical analysis was performed using GraphPad Prism 8.0 software (GraphPad Software Inc, CA, USA). The one-way test ANOVA followed by Tukey's post hoc test was used for normal data, while the Kruskal–Wallis test was used for non-normal data. The results are presented as the mean ± standard error. A P-value of less than 0.05 was considered to be statistically significant.

### Ethics approval and consent to participate

The experimental protocols were established following the Declaration of Helsinki and approved by the ethics committee of Pasteur Institute of Iran (IR.PII.REC1400.061). Signed informed consent was obtained from all participants.

### Supplementary Information

Supplementary Tables.

## Data Availability

The datasets generated during and/or analyzed during the current study are available from the corresponding author on reasonable request.
