# Supplementary material for: The comparative anti-oxidant and anti-inflammatory efficacy of postbiotics and probiotics through Nrf-2 and NF-kB pathways in DSS-induced colitis model
Source: Sci Rep. 2024 May 21;14:11560. doi: 10.1038/s41598-024-62441-0 (PMC11109304; doi:10.1038/s41598-024-62441-0)
Supplement: Supplementary file 1 — Supplementary Tables. [file 41598_2024_62441_MOESM1_ESM.docx]

| Biochemical tests (Mean average%) | | | | | |  |
| --- | --- | --- | --- | --- | --- | --- |
| Strains-probiotic cocktail | DPPH | ABTS | HRS | Superoxide anion | RP | Lipid |
| *L. plantarum* 42 | 72.67±5.50 | 59.33±8.505 | 66.00±5.568 | 58.50±10.60 | 2.953±0.1007 | 64.67±3.786 |
| *L. reuterri* 100 | 69.33±11.93 | 66.67±4.163 | 70.00±10.00 | 68±3.559 | 3.450±0.087 | 65.00±10 |
| *L. plantarum* 119 | 71.33±10.37 | 60.00±10.54 | 59.33±4.16 | 61.00±3.367 | 3.340±0.1249 | 61.33±9.018 |
| *L. plantarum* 155 | 70.33±6.80 | 57.67±10.50 | 57.67±3.05 | 57.75±6.752 | 3.467±0.08 | 61.67±4.50 |
| *B. bifidum* 1001 | 54.67±8.08 | 50.33±6.50 | 50±13.23 | 52.25±3.862 | 3.300±0.1 | 54.00±4.359 |
| *B. longum* 1044 | 52.33±7.76 | 53.00±2.646 | 56.67±4.619 | 50.25±8.958 | 3.40±0.13 | 53.33±12.58 |
| Probiotic Cocktail | 78.33±10.02 | 75.33±7.23 | 74.67±5.859 | 76±8.86 | 3.497±0.20 | 72.67±9.01 |
| Positive control | 90.33±5.13 | 84.22±7.285 | 88.22±1.54 | 98.25±8.42 | .  3.630±0.35 | 90.67±17.47 |
| Negative control | 7.333±0.70 | 13.80±4.59 | 12.00±2 | 15.64±5.46 | 0.24±0.13 | 19.29±7.85 |

Table 2. The antioxidant activity of our selected probiotic strains

| Biochemical tests (Mean average%) | | | | | |  |
| --- | --- | --- | --- | --- | --- | --- |
| *Strains-post biotic cocktail* | DPPH | ABTS | HRS | Superoxide anion | RP | Lipid |
| *L. plantarum* 42 | 78±10.15 | 76.00±7.55 | 76.00±9.64 | 76.00±8.88 | 3.370±0.18 | 74.00±10.44 |
| *L. reuterri* 100 | 77±7.55 | 80±19.92 | 77.33±7.234 | 74.33±6.50 | 3.410±0.16 | 76.00±11.53 |
| *L. plantarum* 119 | 81±11.53 | 78.33±10.07 | 71.33±7.095 | 77±10.54 | 3.397±0.17 | 74±8.71 |
| *L. plantarum* 155 | 77±10.82 | 75±7.81 | 79.67±11.02 | 76.33±13.01 | 3.44±0.13 | 75±8.54 |
| *B. bifidum* 1001 | 79±7.55 | 75.33±9.07 | 73±15.13 | 79±10.54 | 3.340±0.05 | 72.67±3.21 |
| *B. longum* 1044 | 78.67±11.24 | 70.67±8.38 | 71.67±16.26 | 72±17.78 | 3.55±0.1 | 77.33±9.713 |
| postbiotic Cocktail | 83.67±11.59 | 82.67±6.5 | 84.33±5.68 | 86.33±6.65 | 3.65±0.06 | 84.33±9.86 |
| Positive control | 90.33±5.13 | 84.22±7.28 | 88.22±1.54 | 98.25±8.42 | .  3.630±0.35 | 90.67±17.47 |
| Negative control | 7.333±0.7 | 13.8±4.59 | 12±2 | 15.64±5.46 | 0.24±0.13 | 19.29±7.85 |

Table 3. The antioxidant activity of our selected postbiotics
